# Supplementary material for: Neuroimaging supports the representational nature of the earliest human engravings
Source: R Soc Open Sci. 2019 Jul 3;6(7):190086. doi: 10.1098/rsos.190086 (PMC6689598; doi:10.1098/rsos.190086)

**Table S1.** Contextual and descriptive data on early engravings used as visual stimuli. CC: Criss-cross; CH: Cross-hatching; CHB: Cross hatched band; Ch/Qt: Charentian – Quina type; CL: Curved lines; CO: Concentric lines; EUP: Early Upper Paleolithic; FL: Fan-like; HB: Hatched band; LP: Lower Paleolithic; MP: Middle Paleolithic; M: Mousterian; MSA: Middle Stone Age; OES: Ostrich egg shell; PAR: Parallel lines; PM: para-Micoquian; PMK: Parallel marks; SPL: Sub-parallel lines; ZZ: Zigzag.


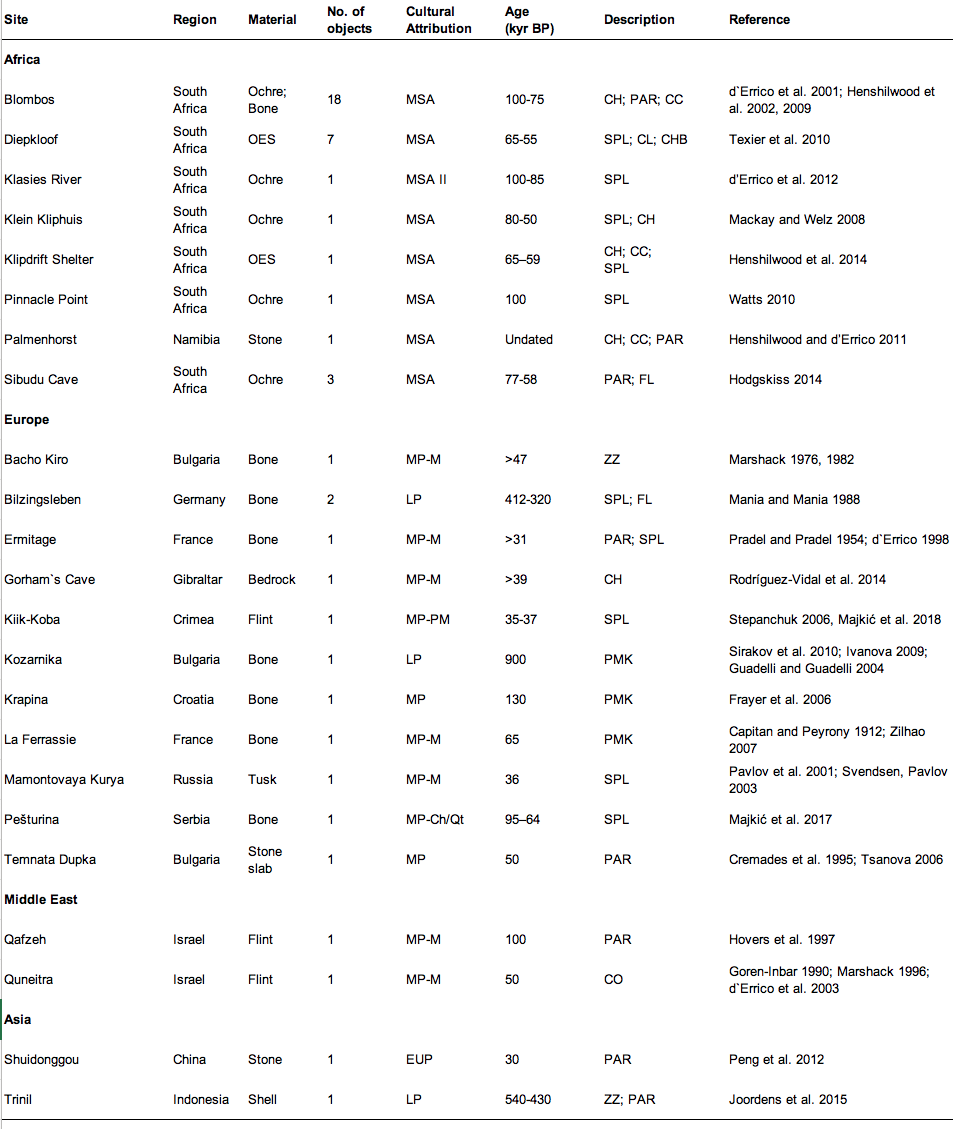

Supplement: Table S1 [file rsos190086supp1.docx]
