# Supplementary material for: Neuroimaging supports the representational nature of the earliest human engravings
Source: R Soc Open Sci. 2019 Jul 3;6(7):190086. doi: 10.1098/rsos.190086 (PMC6689598; doi:10.1098/rsos.190086)
Supplement: Table S2 [file rsos190086supp2.docx]

**Table S2.** Abbreviations and MNI Coordinates of the mass center of the ten hROIs of the Aicha atlas activated by the perception of engravings *minus* scramble contrast

| **hROI** | **Abreviation** | **Left hemisphere** | **Right hemisphere** |
| --- | --- | --- | --- |
|  |  | MNI coordinates | MNI coordinates |
| G_Fusiform-2 | FUS2 | -35 -26 -23 | 38 -25 -24 |
| G_Fusiform-3 | FUS3 | -37 -32 -24 | 37 -31 -24 |
| G_Fusiform-4 | FUS4 | -43 -50 -17 | 44 -46 -18 |
| G_Temporal_Inf-4 | T3-4 | -50 -61 -8 | 54 -58 -11 |
| G_Fusiform-5 | FUS5 | -31 -50 -12 | 32 -47 -11 |
| G_Temporal_Inf-5 | T3-5 | -45 -64 6 | 49 -58 4 |
| G_Occipital_Inf-1 | O3-1 | -48 -69 –4 | 50 -60 -9 |
| G_Occipital_Inf-2 | O3-2 | -45 -71 -7 | 47 -65 -7 |
| G_Occipital_Mid-2 | O2-2 | -37 -77 14 | 41 -73 12 |
| G_Occipital_Lat-3 | OLat3 | -40 -84 -12 | 43 -81 -12 |
