# Supplementary material for: Neuroimaging supports the representational nature of the earliest human engravings
Source: R Soc Open Sci. 2019 Jul 3;6(7):190086. doi: 10.1098/rsos.190086 (PMC6689598; doi:10.1098/rsos.190086)
Supplement: Table S3 [file rsos190086supp3.docx]

**Table S3:** Mean BOLD value of the hROIs activated in the objects *minus* scrambled object contrast (p< 0.05 uncorrected)

| **Activation of objects minus scrambled objects** | | | | | | | | |
| --- | --- | --- | --- | --- | --- | --- | --- | --- |
|  | Left hemisphere | | | | Right hemisphere | | | |
|  | Mean BOLD | SD | t | p | Mean BOLD | SD | t | p |
| G_Fusiform-1 | 0.15 | 0.02 | 6.25 | <.0001 | 0.10 | 0.03 | 3.96 | 0.0005 |
| G_Fusiform-2 | 0.19 | 0.03 | 6.33 | <.0001 | 0.17 | 0.03 | 5.09 | <.0001 |
| G_Fusiform-3 | 0.19 | 0.04 | 4.86 | <.0001 | 0.10 | 0.03 | 3.26 | 0.0032 |
| G_Fusiform-4 | 0.43 | 0.07 | 6.29 | <.0001 | 0.25 | 0.05 | 4.62 | 0.0001 |
| G_Fusiform-5 | 0.34 | 0.04 | 8.09 | <.0001 | 0.25 | 0.04 | 6.35 | <.0001 |
| G_Fusiform-6 | 0.10 | 0.04 | 2.25 | 0.0332 | 0.14 | 0.05 | 3.04 | 0.0054 |
| G_Occipital_Inf-1 | 0.55 | 0.08 | 6.72 | <.0001 | 0.16 | 0.07 | 2.22 | 0.0360 |
| G_Occipital_Inf-2 | 0.52 | 0.07 | 7.81 | <.0001 | 0.41 | 0.06 | 6.44 | <.0001 |
| G_Occipital_Lat-3 | 0.37 | 0.08 | 4.81 | <.0001 | 0.48 | 0.10 | 4.89 | <.0001 |
| G_Occipital_Lat-5 | 0.15 | 0.05 | 2.89 | 0.0078 | 0.14 | 0.05 | 2.76 | 0.0106 |
| G_Occipital_Mid-2 | 0.11 | 0.05 | 2.15 | 0.0416 | 0.15 | 0.05 | 3.15 | 0.0042 |
| G_Occipital_Mid-3 | 0.19 | 0.05 | 3.90 | 0.0006 | 0.15 | 0.04 | 3.65 | 0.0012 |
| G_ParaHippocampal-2 | 0.25 | 0.04 | 6.04 | <.0001 | 0.16 | 0.03 | 5.43 | <.0001 |
| G_ParaHippocampal-5 |  |  |  |  | 0.15 | 0.03 | 4.51 | 0.0001 |
| G_Temporal_Inf-4 | 0.37 | 0.07 | 5.07 | <.0001 |  |  |  |  |
| G_Temporal_Inf-5 | 0.26 | 0.05 | 5.04 | <.0001 | 0.12 | 0.05 | 2.53 | 0.0182 |
| G_Temporal_Mid-4 | 0.26 | 0.06 | 4.34 | 0.0002 |  |  |  |  |
| S_Orbital-2 |  |  |  |  | 0.14 | 0.04 | 3.23 | 0.0035 |
