# Supplementary material for: Neuroimaging supports the representational nature of the earliest human engravings
Source: R Soc Open Sci. 2019 Jul 3;6(7):190086. doi: 10.1098/rsos.190086 (PMC6689598; doi:10.1098/rsos.190086)
Supplement: Table S4 [file rsos190086supp4.docx]

**Table S4:** Mean BOLD value of the hROIs activated in the scenes *minus* scrambled scenes contrast (p< 0.05 uncorrected)

| **Activation of scene minus scrambled scene** | | | | | | | | | | |
| --- | --- | --- | --- | --- | --- | --- | --- | --- | --- | --- |
|  | Left hemisphere | | | | | Right hemisphere | | | | |
|  | Mean BOLD | | SD | t | p | Mean BOLD | | SD | t | p |
| S_Orbital-2 |  |  | |  |  | 0.12 | 0.03 | | 3.82 | 0.0008 |
| G_Occipital_Lat-3 |  |  | |  |  | 0.14 | 0.06 | | 2.15 | 0.0411 |
| G_Occipital_Lat-5 | 0.12 | 0.04 | | 3.05 | 0.0054 | 0.18 | 0.04 | | 4.56 | 0.0001 |
| G_Occipital_Mid-1 | 0.31 | 0.05 | | 6.69 | <.0001 | 0.28 | 0.05 | | 5.69 | <.0001 |
| G_Occipital_Mid-2 | 0.21 | 0.04 | | 5.71 | <.0001 | 0.20 | 0.04 | | 5.40 | <.0001 |
| G_Occipital_Mid-3 | 0.25 | 0.04 | | 6.74 | <.0001 | 0.24 | 0.04 | | 6.78 | <.0001 |
| G_Occipital_Mid-4 | 0.50 | 0.06 | | 9.05 | <.0001 | 0.43 | 0.06 | | 7.49 | <.0001 |
| G_Occipital_Inf-2 | 0.12 | 0.04 | | 2.92 | 0.0072 | 0.15 | 0.05 | | 3.27 | 0.0032 |
| G_Lingual-1 | 0.51 | 0.05 | | 10.28 | <.0001 | 0.43 | 0.04 | | 10.03 | <.0001 |
| G_Lingual-2 | 0.23 | 0.05 | | 4.87 | <.0001 | 0.24 | 0.04 | | 5.77 | <.0001 |
| G_Fusiform-2 | 0.07 | 0.02 | | 2.89 | 0.0079 | 0.12 | 0.02 | | 5.40 | <.0001 |
| G_Fusiform-3 | 0.06 | 0.03 | | 2.14 | 0.0421 | 0.10 | 0.03 | | 3.91 | 0.0006 |
| G_Fusiform-4 | 0.10 | 0.05 | | 2.11 | 0.0455 | 0.11 | 0.04 | | 3.04 | 0.0055 |
| G_Fusiform-5 | 0.31 | 0.04 | | 7.09 | <.0001 | 0.44 | 0.05 | | 9.48 | <.0001 |
| G_Fusiform-6 | 0.10 | 0.04 | | 2.65 | 0.0139 | 0.21 | 0.04 | | 4.78 | <.0001 |
| G_ParaHippocampal-1 |  |  | |  |  | 0.21 | 0.05 | | 3.87 | 0.0007 |
| G_ParaHippocampal-2 | 0.30 | 0.04 | | 8.03 | <.0001 | 0.29 | 0.04 | | 6.52 | <.0001 |
| G_ParaHippocampal-4 | 0.33 | 0.05 | | 6.46 | <.0001 | 0.33 | 0.05 | | 5.99 | <.0001 |
| G_ParaHippocampal-5 | 0.56 | 0.05 | | 10.66 | <.0001 | 0.53 | 0.04 | | 12.13 | <.0001 |
| G_Temporal_Mid-4 |  |  | |  |  | 0.13 | 0.04 | | 2.83 | 0.0090 |
| G_Temporal_Inf-5 |  |  | |  |  | 0.09 | 0.03 | | 2.89 | 0.0078 |
| N_Thalamus-9 | 0.12 | 0.05 | | 2.34 | 0.0276 | 0.16 | 0.04 | | 4.44 | 0.0002 |
| S_Sup_Temporal-4 |  |  | |  |  |  |  | |  |  |
