# Supplementary material for: Neuroimaging supports the representational nature of the earliest human engravings
Source: R Soc Open Sci. 2019 Jul 3;6(7):190086. doi: 10.1098/rsos.190086 (PMC6689598; doi:10.1098/rsos.190086)
Supplement: Table S5 [file rsos190086supp5.docx]

**Table S5:** Mean BOLD value of the hROIs activated in the linear B *minus* scrambled linear B contrast (p< 0.05 uncorrected)

| **Activation of linearB minus scrambled linearB** | | | | | | | | | |
| --- | --- | --- | --- | --- | --- | --- | --- | --- | --- |
|  | Left hemisphere | | | | | Right hemisphere | | | |
|  | Mean BOLD | | SD | t | p | Mean BOLD | SD | t | p |
| S_Orbital-1 |  |  | |  |  | 0.10 | 0.04 | 2.62 | 0.0146 |
| S_Sup_Frontal-5 |  |  | |  |  | 0.15 | 0.05 | 2.71 | 0.0120 |
| S_Inf_Frontal-1 | 0.17 | 0.08 | | 2.08 | 0.0482 |  |  |  |  |
| G_Frontal_Sup-3 | 0.17 | 0.05 | | 3.72 | 0.0010 |  |  |  |  |
| G_Frontal_Mid-5 | 0.21 | 0.07 | | 2.91 | 0.0075 | 0.18 | 0.07 | 2.58 | 0.0163 |
| G_Frontal_Mid-4 |  |  | |  |  | 0.23 | 0.08 | 2.91 | 0.0076 |
| G_Frontal_Mid_Orb-1 |  |  | |  |  | 0.22 | 0.09 | 2.47 | 0.0209 |
| G_Frontal_Mid_Orb-2 | 0.31 | 0.08 | | 3.80 | 0.0008 | 0.22 | 0.08 | 2.79 | 0.0100 |
| G_Frontal_Sup_Medial-3 | 0.18 | 0.05 | | 3.32 | 0.0028 | 0.18 | 0.06 | 3.04 | 0.0055 |
| G_Occipital_Inf-1 | 0.19 | 0.09 | | 2.18 | 0.0388 |  |  |  |  |
| G_Occipital_Inf-2 | 0.33 | 0.07 | | 4.94 | <.0001 | 0.26 | 0.06 | 4.25 | 0.0003 |
| G_Occipital_Lat-3 | 0.32 | 0.08 | | 3.81 | 0.0008 | 0.42 | 0.10 | 4.21 | 0.0003 |
| G_Occipital_Lat-5 | 0.12 | 0.06 | | 2.24 | 0.0344 |  |  |  |  |
| G_Lingual-5 | 0.16 | 0.07 | | 2.16 | 0.0408 |  |  |  |  |
| G_Fusiform-3 | 0.14 | 0.04 | | 3.67 | 0.0012 | 0.11 | 0.03 | 4.24 | 0.0003 |
| G_Fusiform-4 | 0.40 | 0.07 | | 5.65 | <.0001 | 0.23 | 0.04 | 5.28 | <.0001 |
| G_Fusiform-5 | 0.14 | 0.04 | | 3.17 | 0.0040 |  |  |  |  |
| G_Fusiform-6 | 0.14 | 0.04 | | 3.39 | 0.0023 |  |  |  |  |
| G_Fusiform-7 | 0.14 | 0.06 | | 2.47 | 0.0207 |  |  |  |  |
| G_Temporal_Mid-2 |  |  | |  |  | 0.17 | 0.05 | 3.55 | 0.0016 |
| G_Temporal_Inf-3 | 0.21 | 0.07 | | 3.13 | 0.0045 |  |  |  |  |
| G_Parietal_Inf-1 | 0.31 | 0.09 | | 3.50 | 0.0018 | 0.32 | 0.09 | 3.56 | 0.0015 |
| G_SupraMarginal-6 | 0.24 | 0.09 | | 2.65 | 0.0138 | 0.20 | 0.09 | 2.24 | 0.0339 |
| G_SupraMarginal-7 |  |  | |  |  | 0.27 | 0.09 | 3.11 | 0.0046 |
| G_Angular-1 |  |  | |  |  | 0.38 | 0.09 | 4.10 | 0.0004 |
| N_Caudate-5 | 0.19 | 0.07 | | 2.61 | 0.0150 | 0.21 | 0.08 | 2.83 | 0.0090 |
| N_Thalamus-2 | 0.14 | 0.07 | | 2.11 | 0.0451 |  |  |  |  |
