# Supplementary material for: Neuroimaging supports the representational nature of the earliest human engravings
Source: R Soc Open Sci. 2019 Jul 3;6(7):190086. doi: 10.1098/rsos.190086 (PMC6689598; doi:10.1098/rsos.190086)
Supplement: Table S6 [file rsos190086supp6.docx]

**Table S6:** Mean BOLD value of the hROIs activated in the Words *minus* scrambled words contrast (p< 0.05 uncorrected)

| **Activation of words minus scrambled words** | | | | | | | | |
| --- | --- | --- | --- | --- | --- | --- | --- | --- |
|  | Left hemisphere | | | | Right hemisphere | | | |
|  | Mean BOLD | SD | t | p | Mean BOLD | SD | t | p |
| G_Fusiform-1 | 0.11 | 0.02 | 4.40 | 0.0002 |  |  |  |  |
| G_Fusiform-2 | 0.07 | 0.03 | 2.27 | 0.0323 |  |  |  |  |
| G_Fusiform-4 | 0.25 | 0.06 | 4.22 | 0.0003 |  |  |  |  |
| G_Temporal_Sup-4 | 0.19 | 0.04 | 4.42 | 0.0002 |  |  |  |  |
| G_Temporal_Mid-4 | 0.14 | 0.04 | 3.21 | 0.0036 |  |  |  |  |
| G_Temporal_Inf-3 | 0.18 | 0.05 | 3.50 | 0.0018 |  |  |  |  |
| S_Sup_Temporal-4 | 0.32 | 0.05 | 6.13 | <.0001 |  |  |  |  |
| S_Sup_Temporal-3 |  |  |  |  | 0.19 | 0.05 | 3.48 | 0.0019 |
