# Supplementary figures and images for: Neuroimaging supports the representational nature of the earliest human engravings
Source: R Soc Open Sci. 2019 Jul 3;6(7):190086. doi: 10.1098/rsos.190086 (PMC6689598; doi:10.1098/rsos.190086)

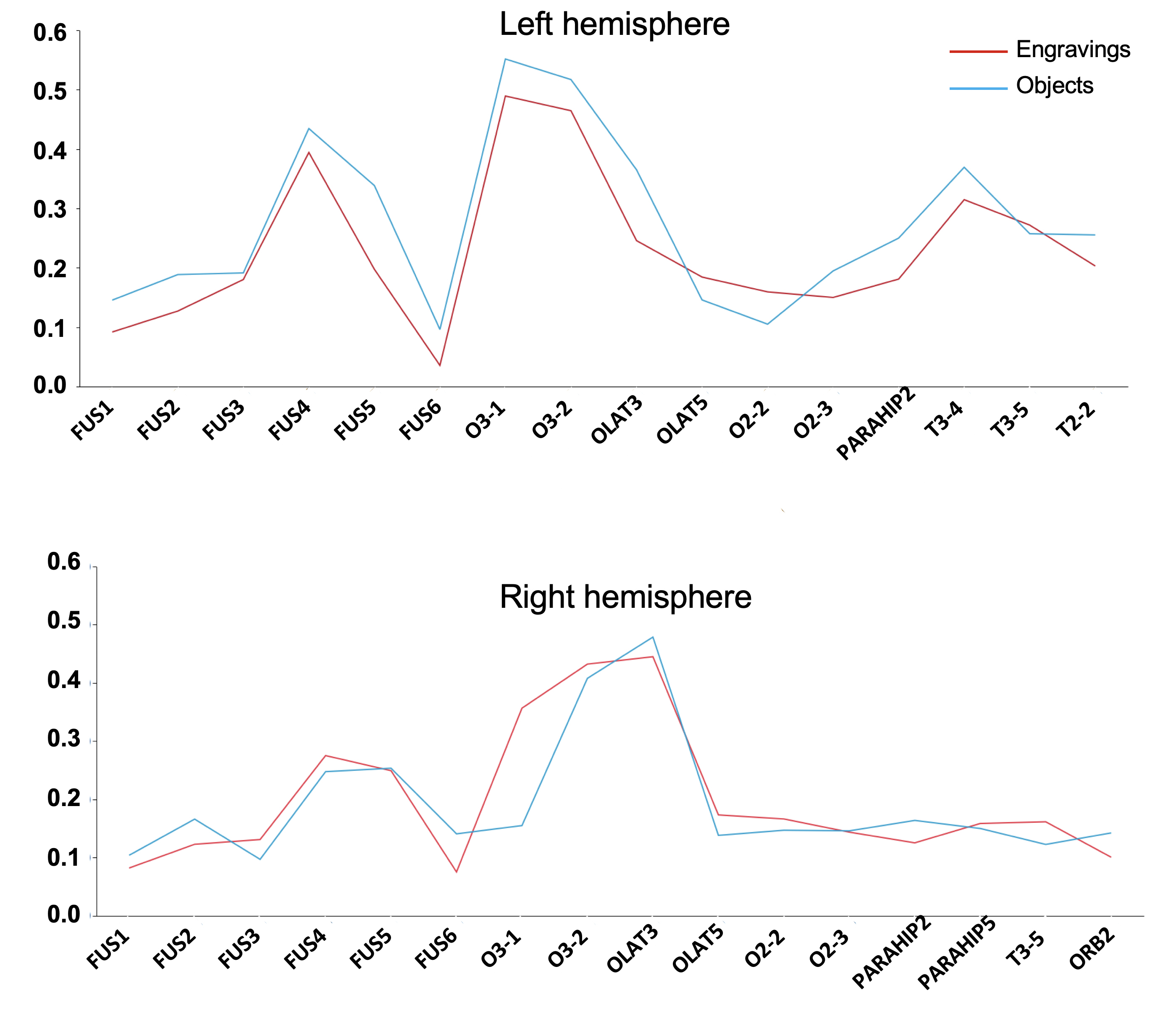

Supplement: Figure S1 [file rsos190086supp7.jpg]
