# Supplementary material for: Neuroimaging supports the representational nature of the earliest human engravings
Source: R Soc Open Sci. 2019 Jul 3;6(7):190086. doi: 10.1098/rsos.190086 (PMC6689598; doi:10.1098/rsos.190086)
Supplement: Figure S2 [file rsos190086supp8.pdf]

Engravings

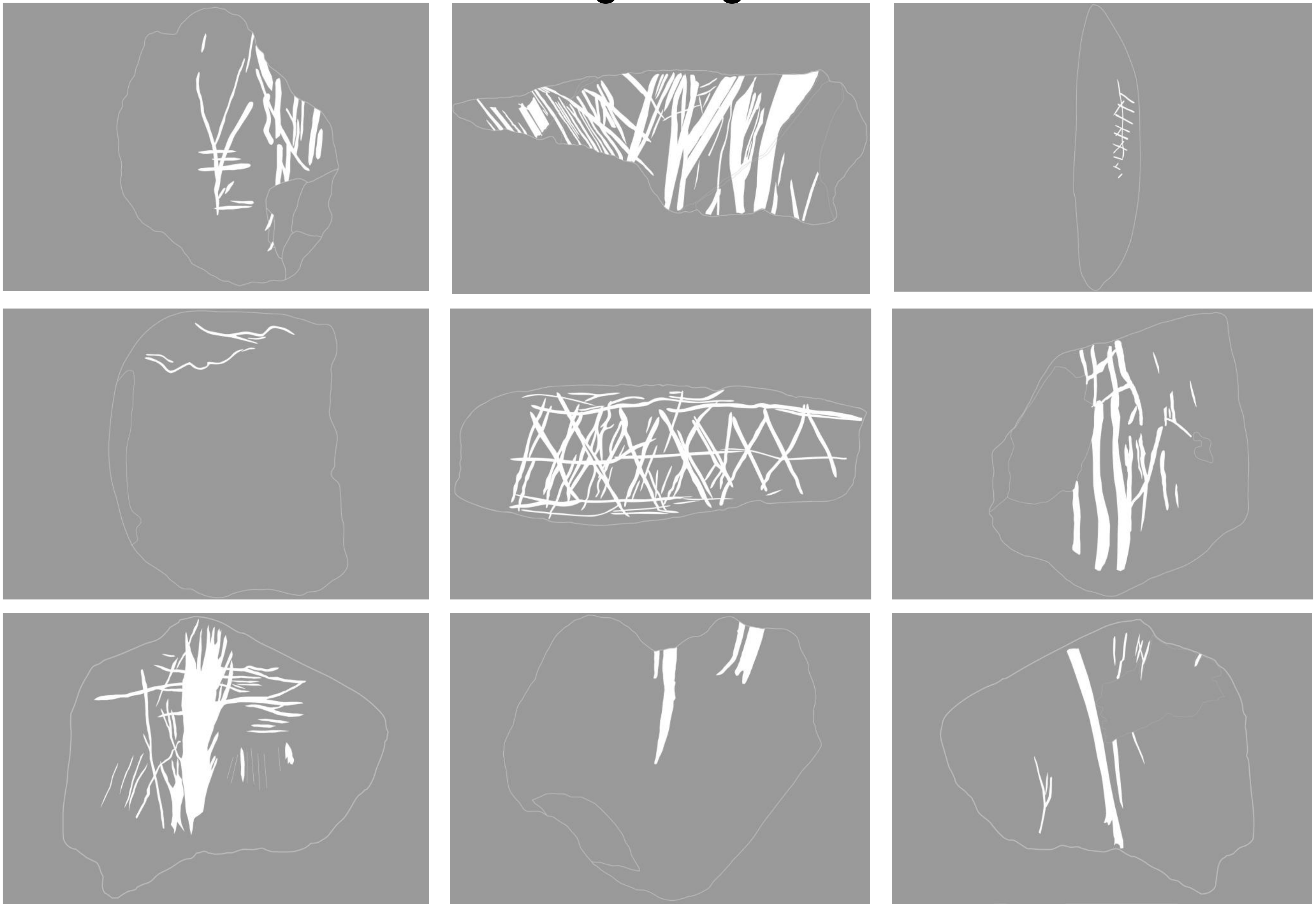

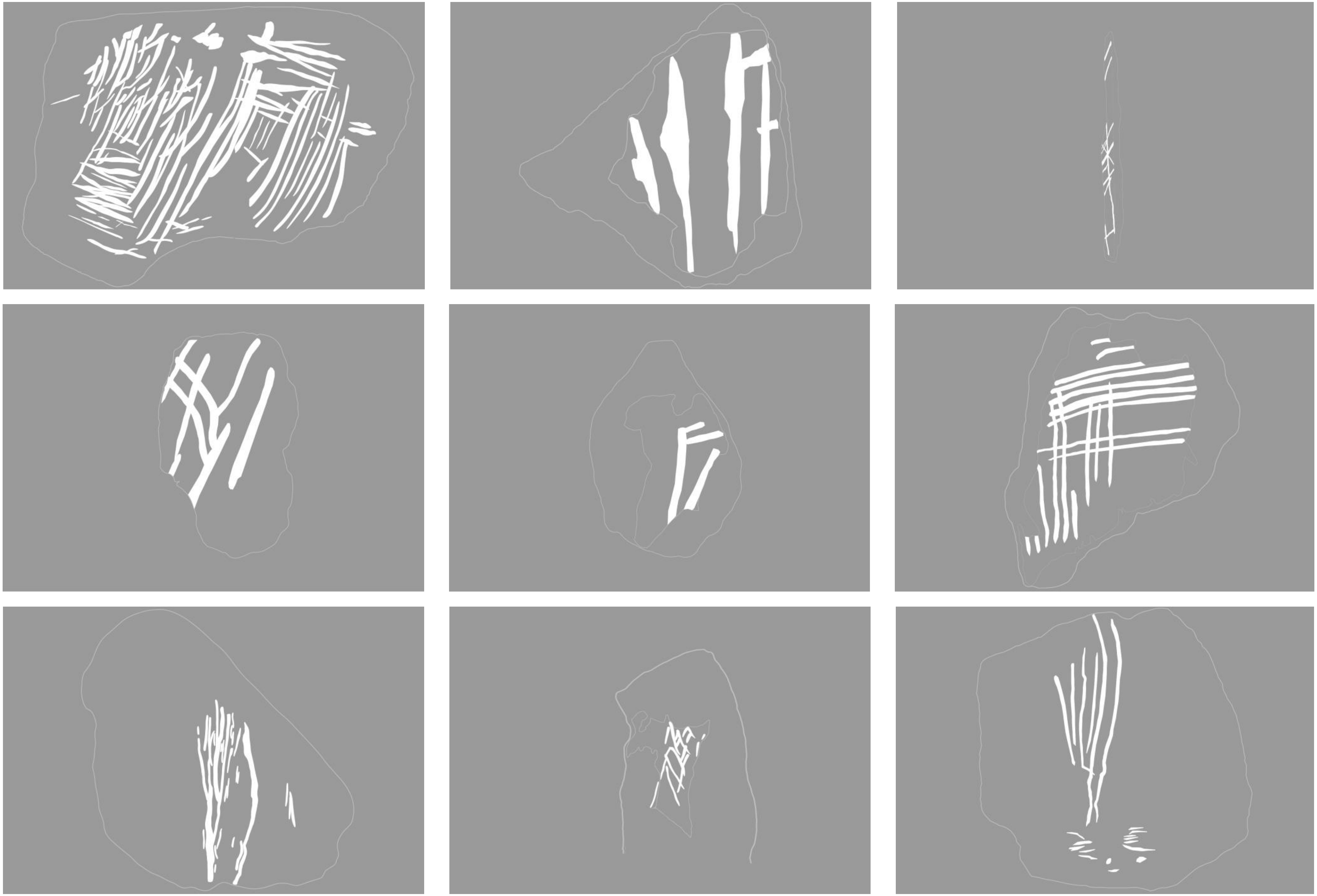

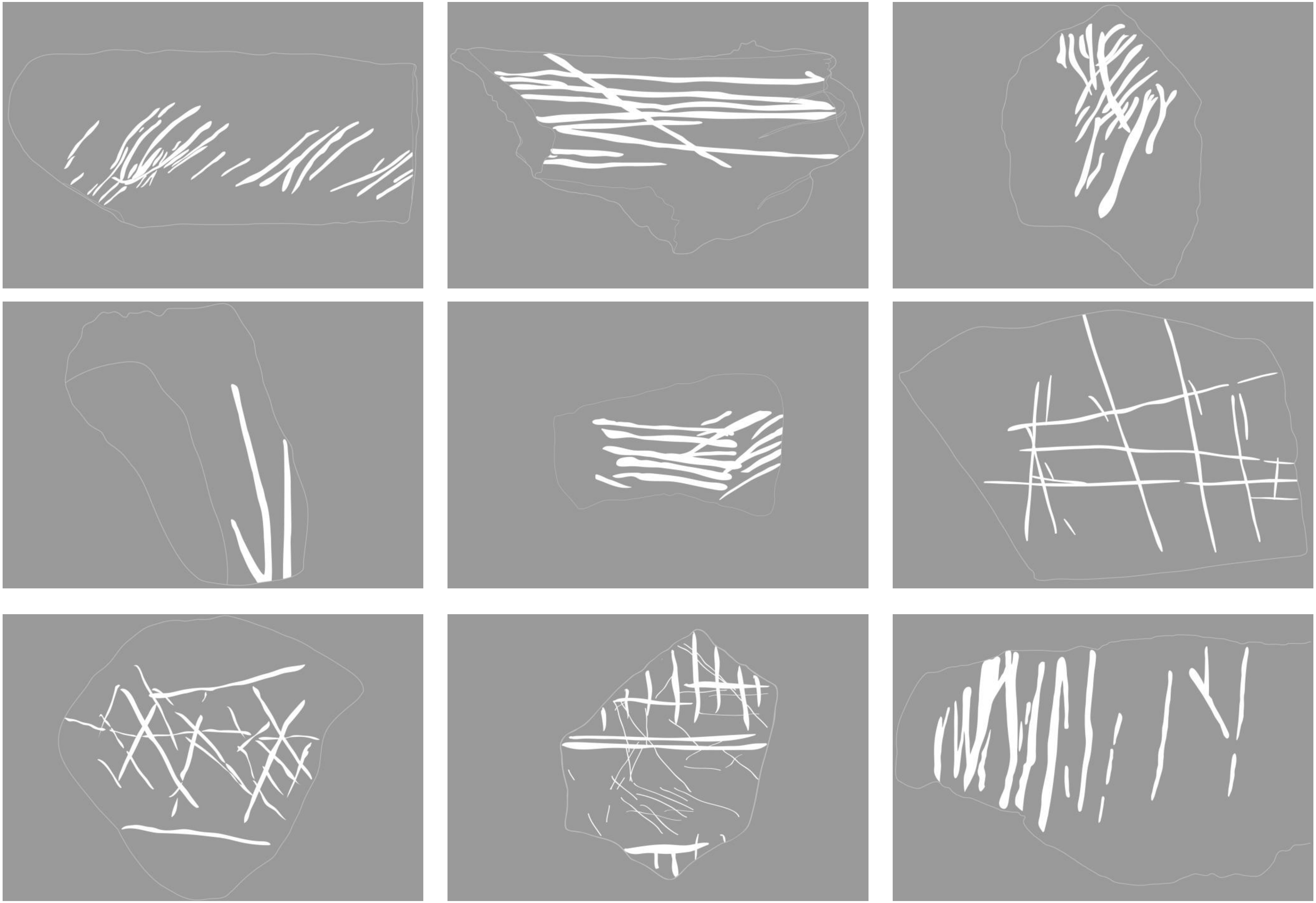

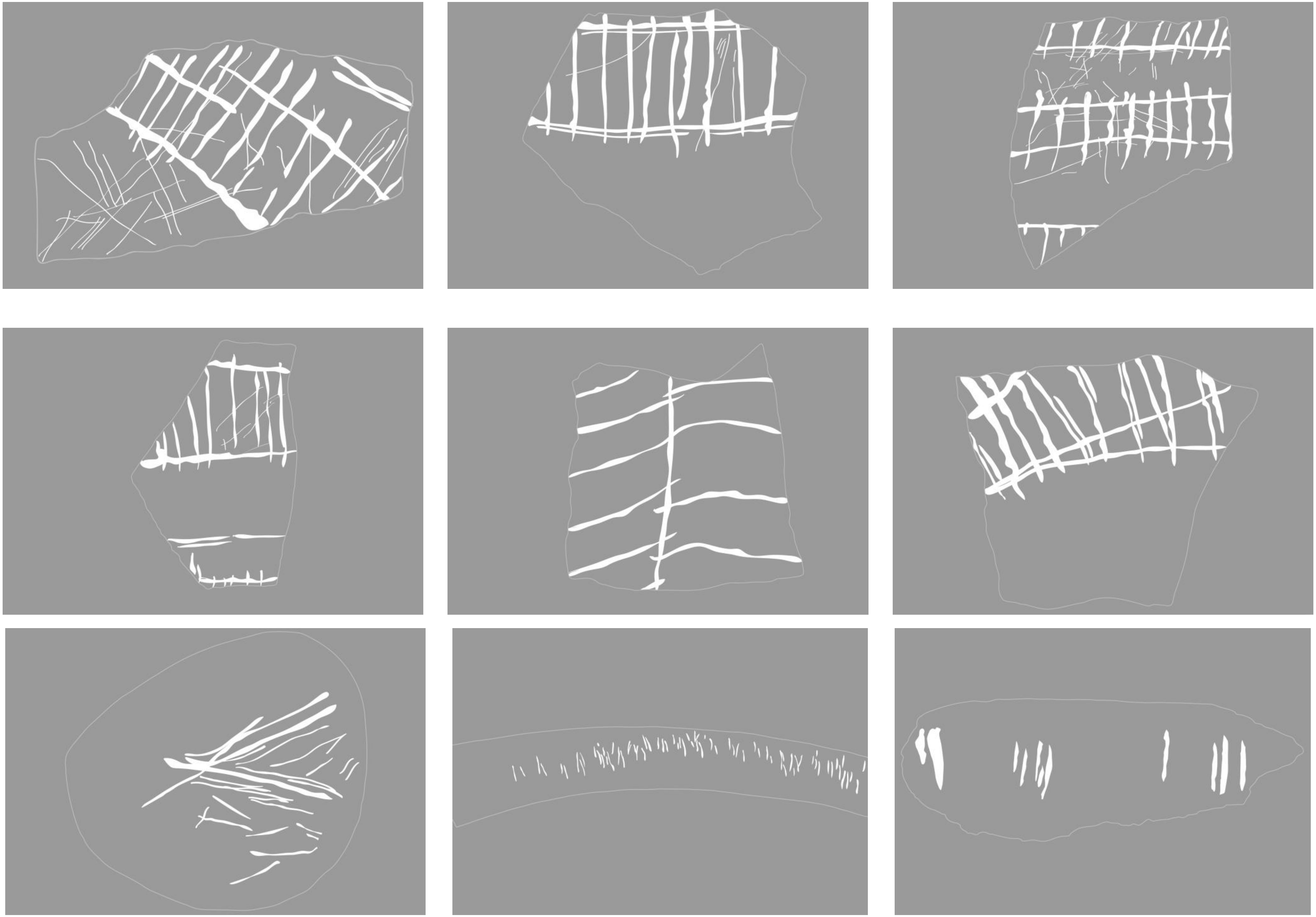

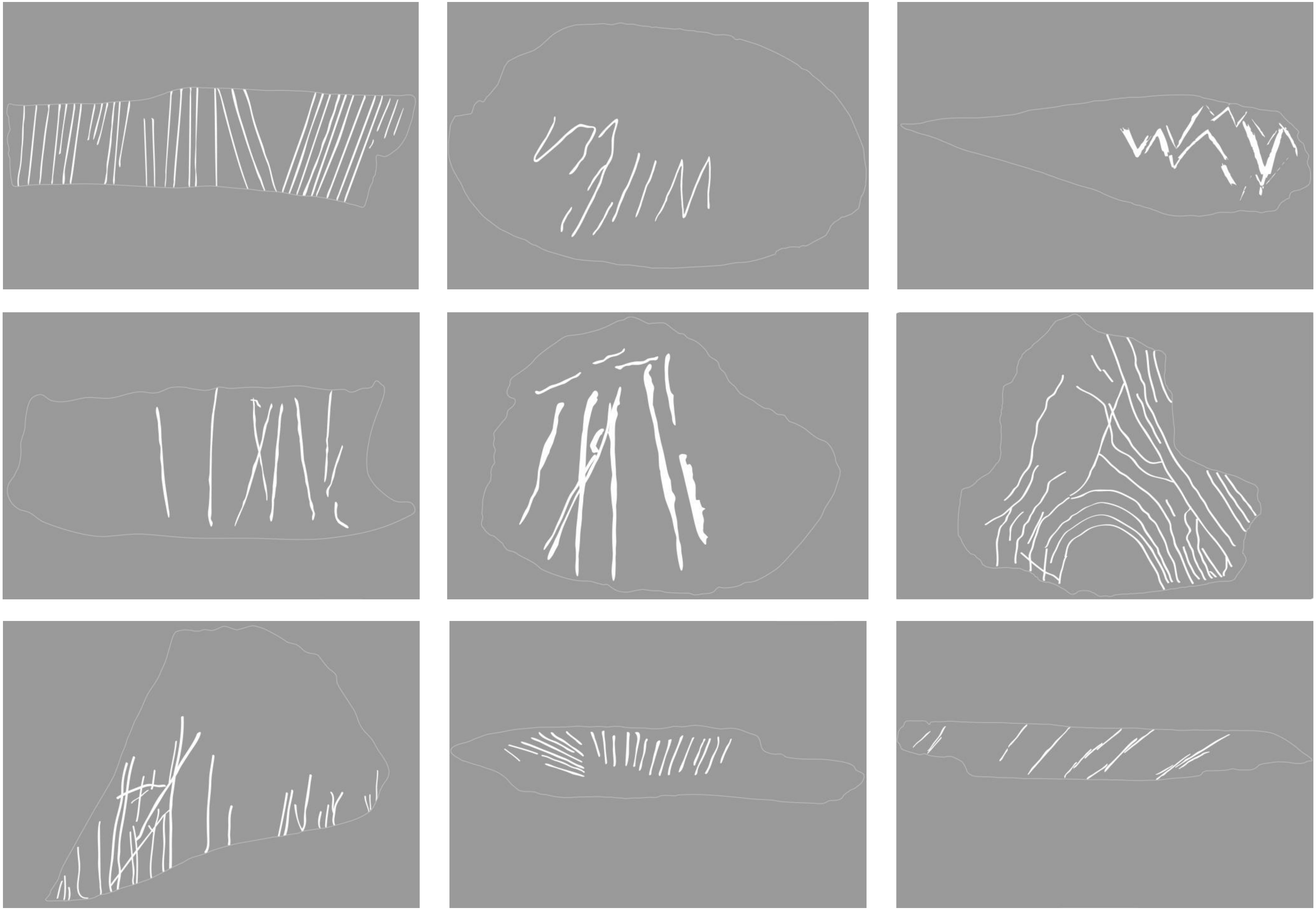

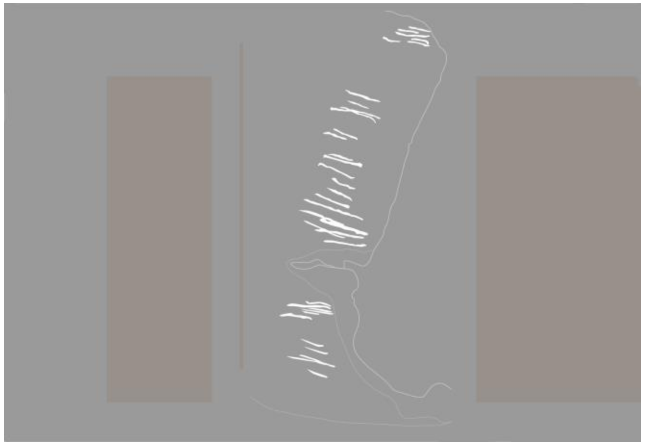

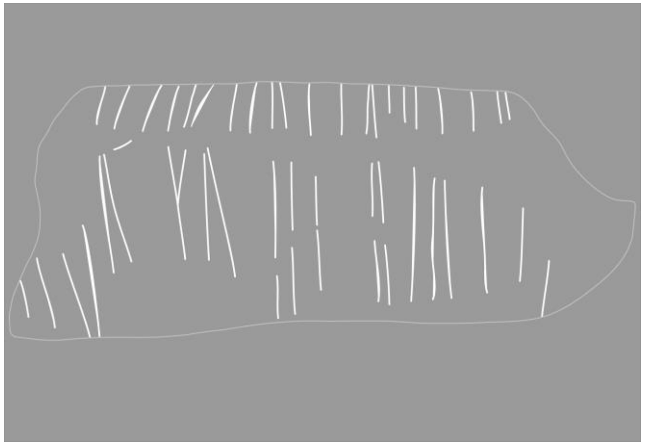

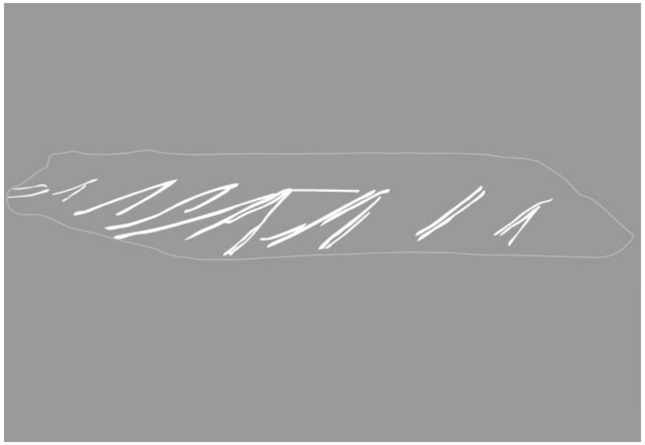

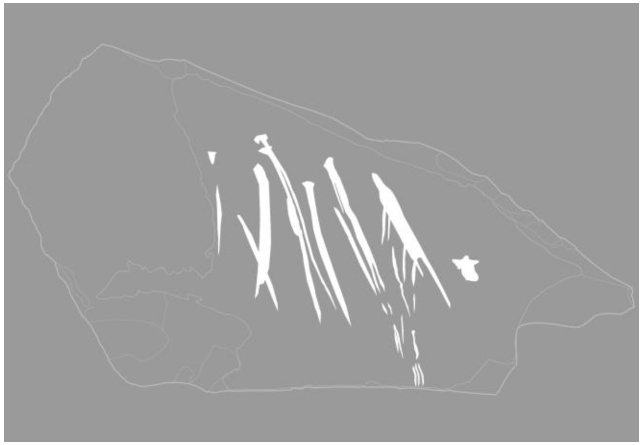

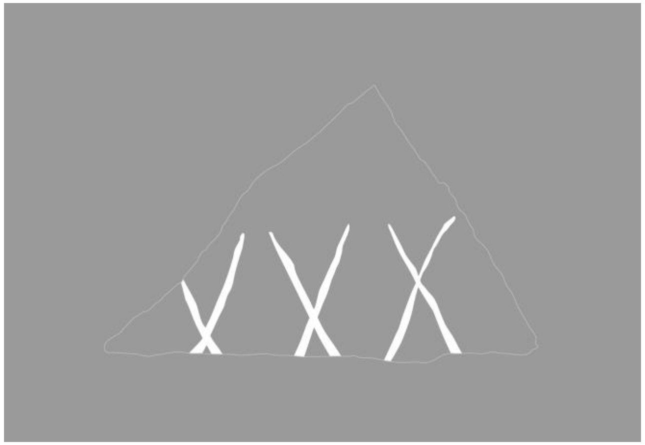

Objects

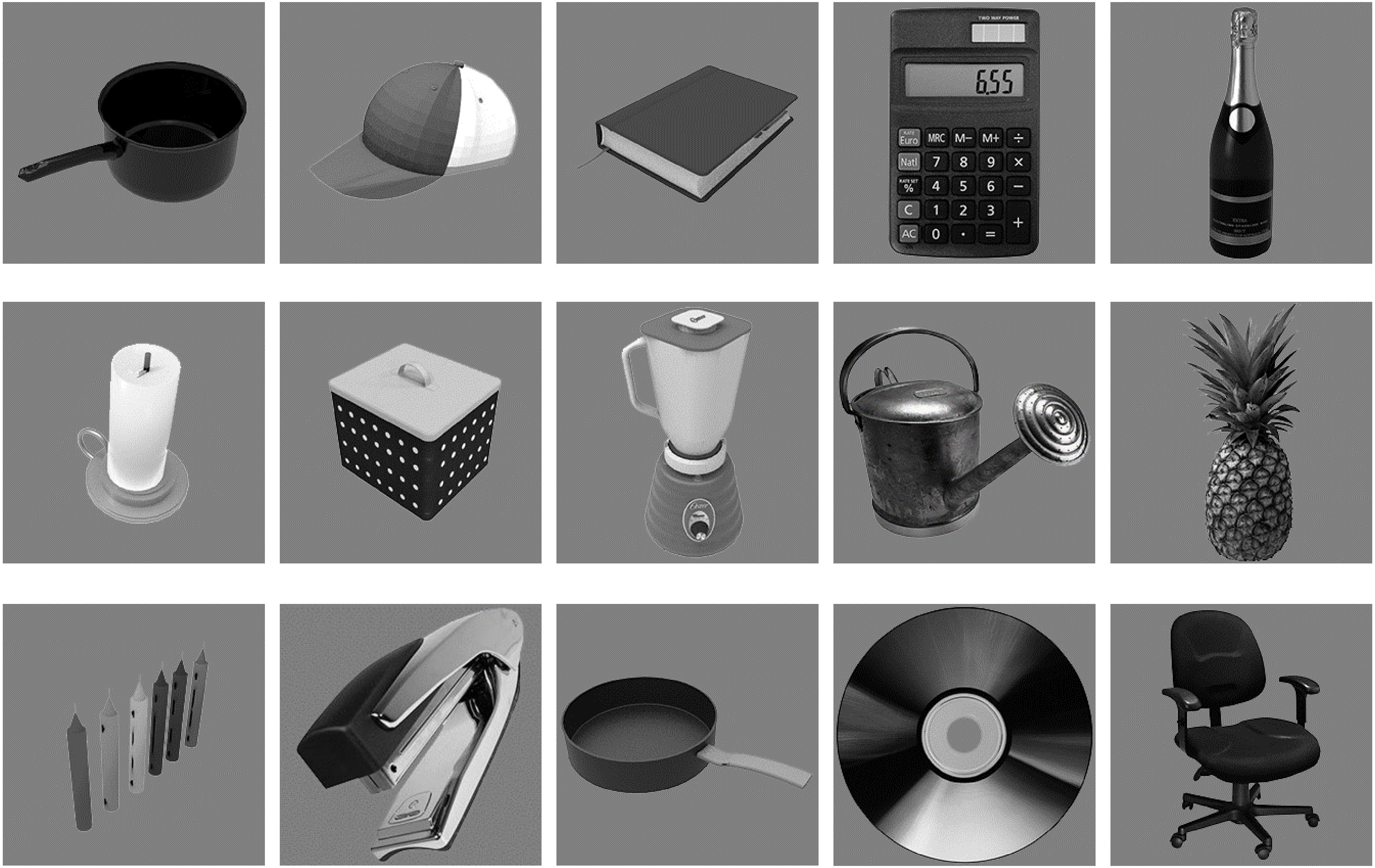

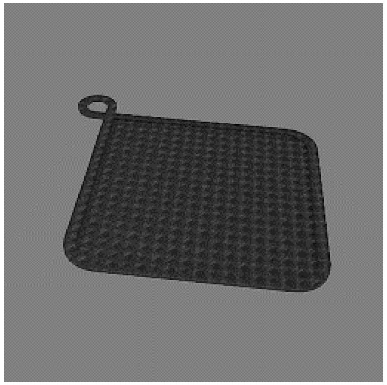
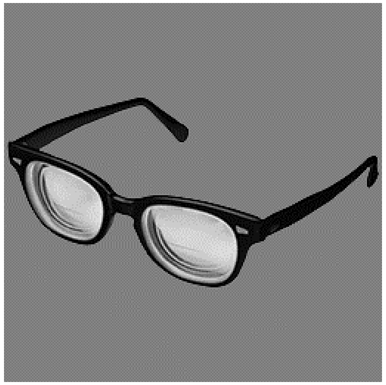
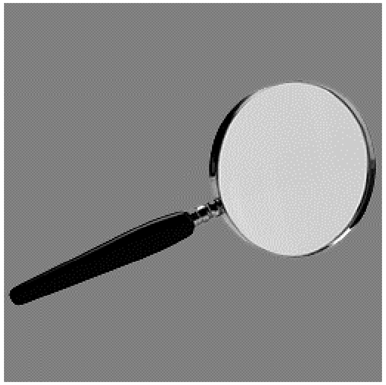
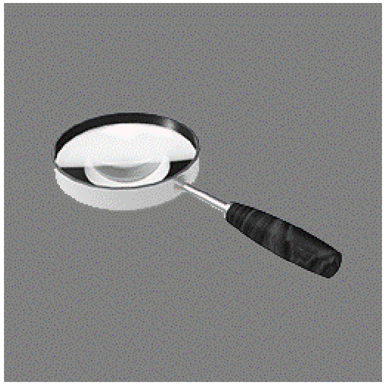
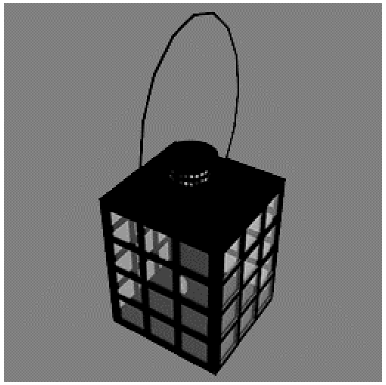
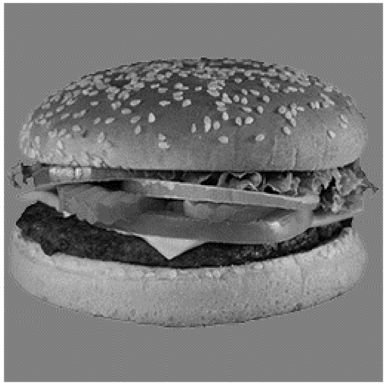
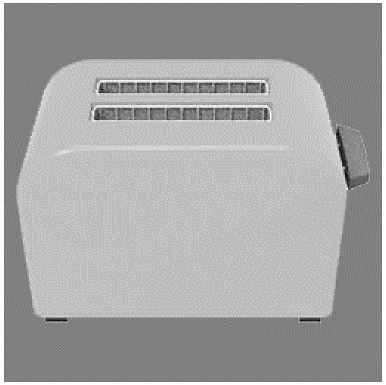
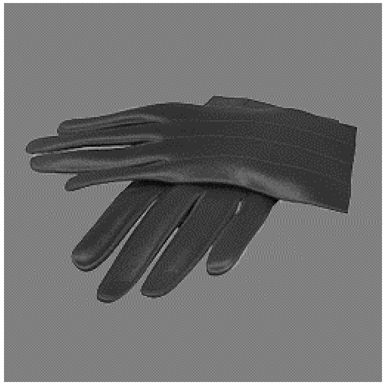
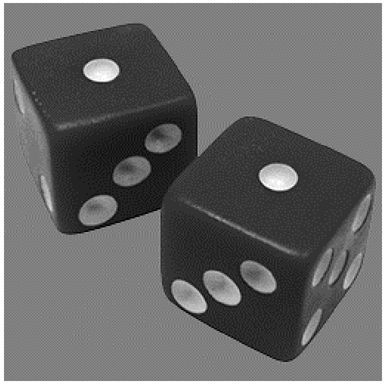
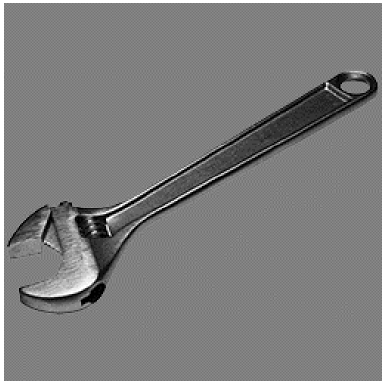
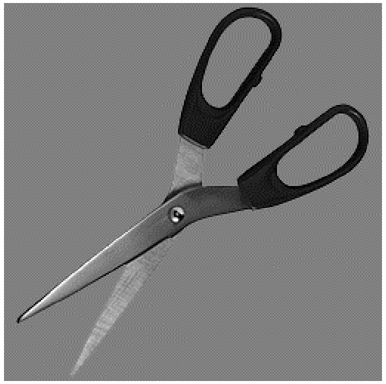
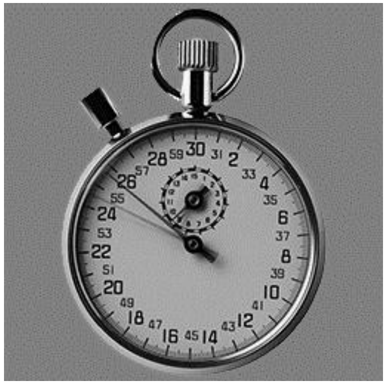
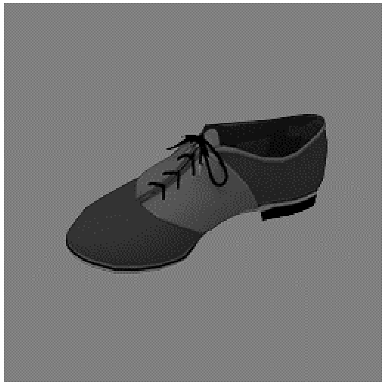
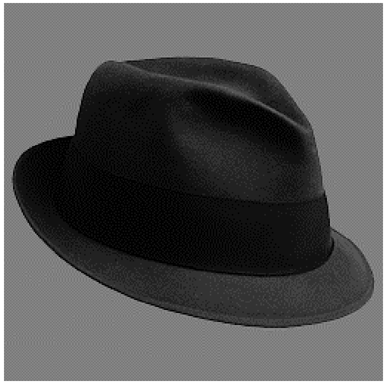
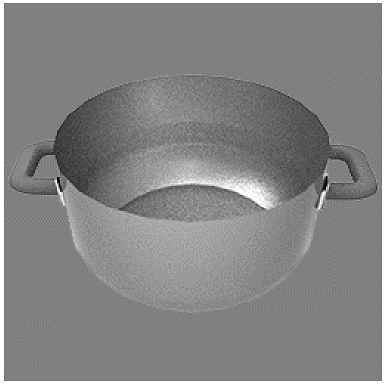

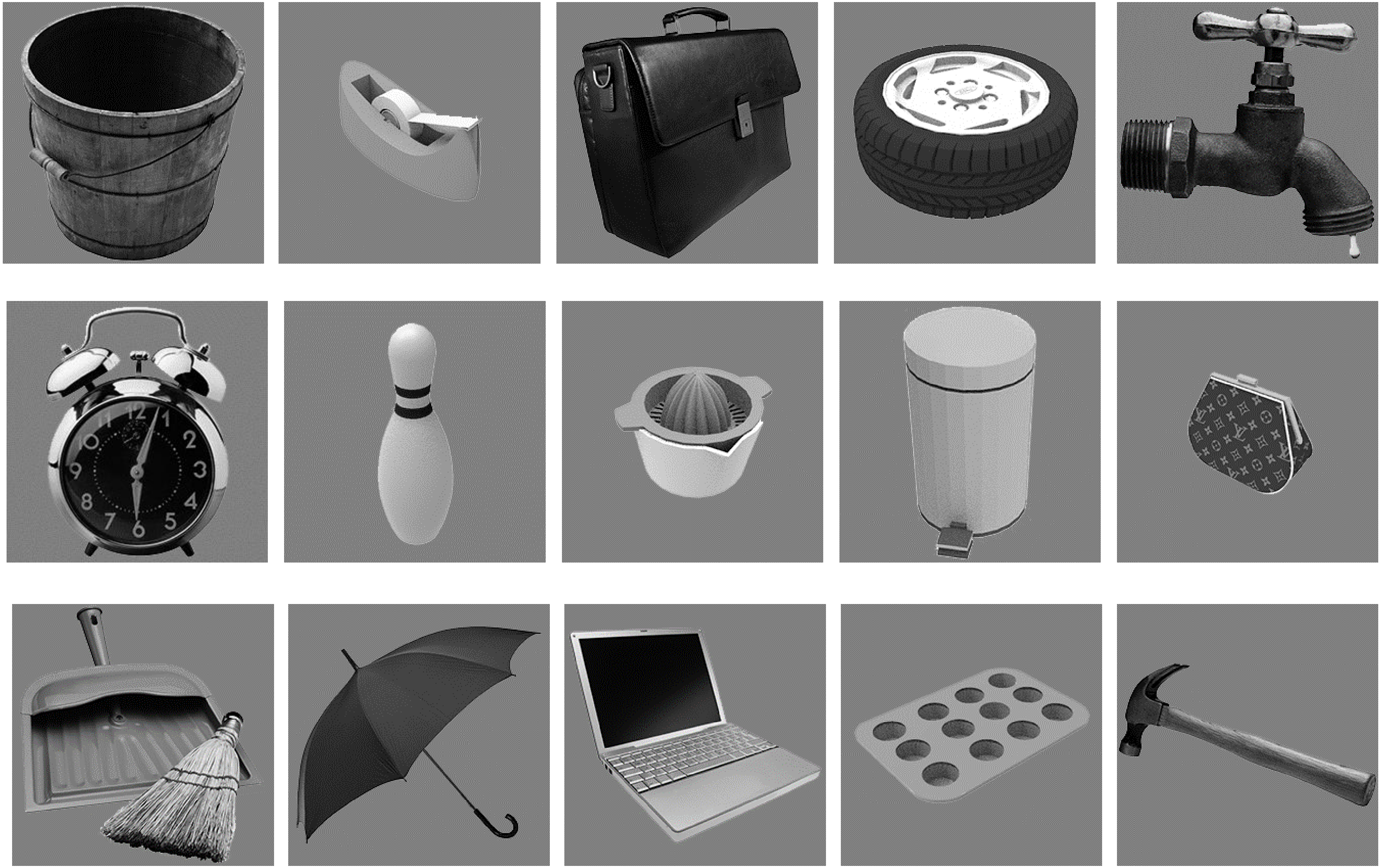

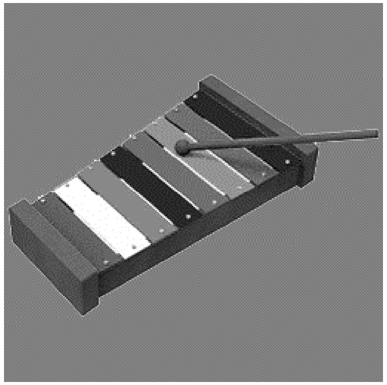
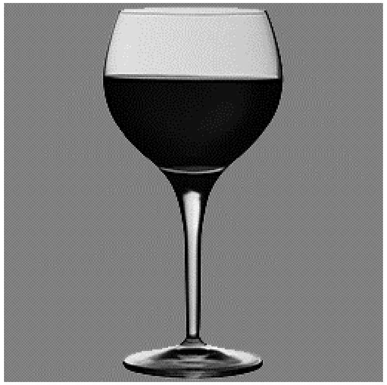
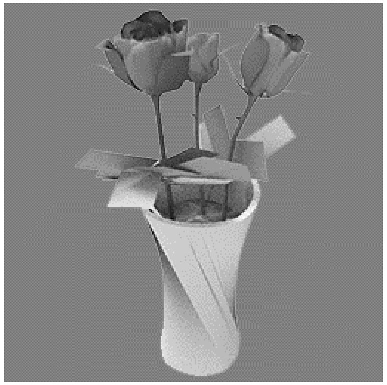
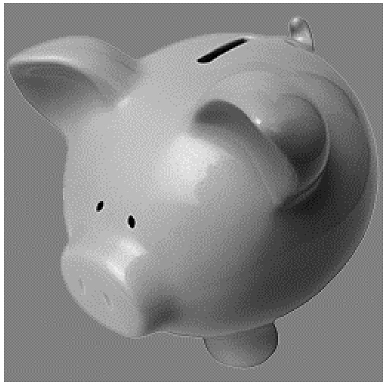
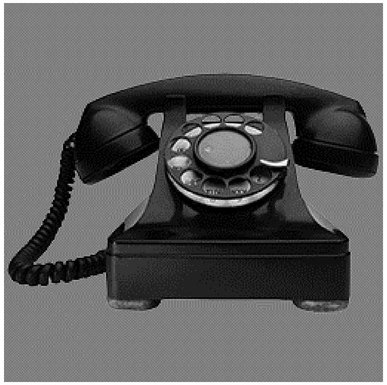
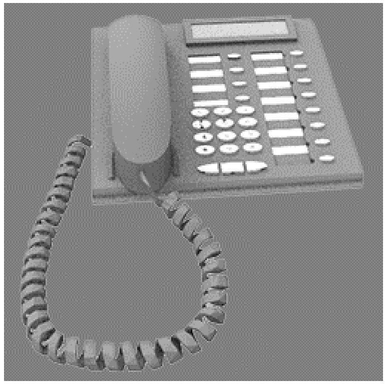
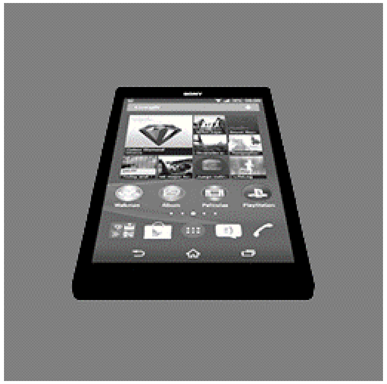

Scenes

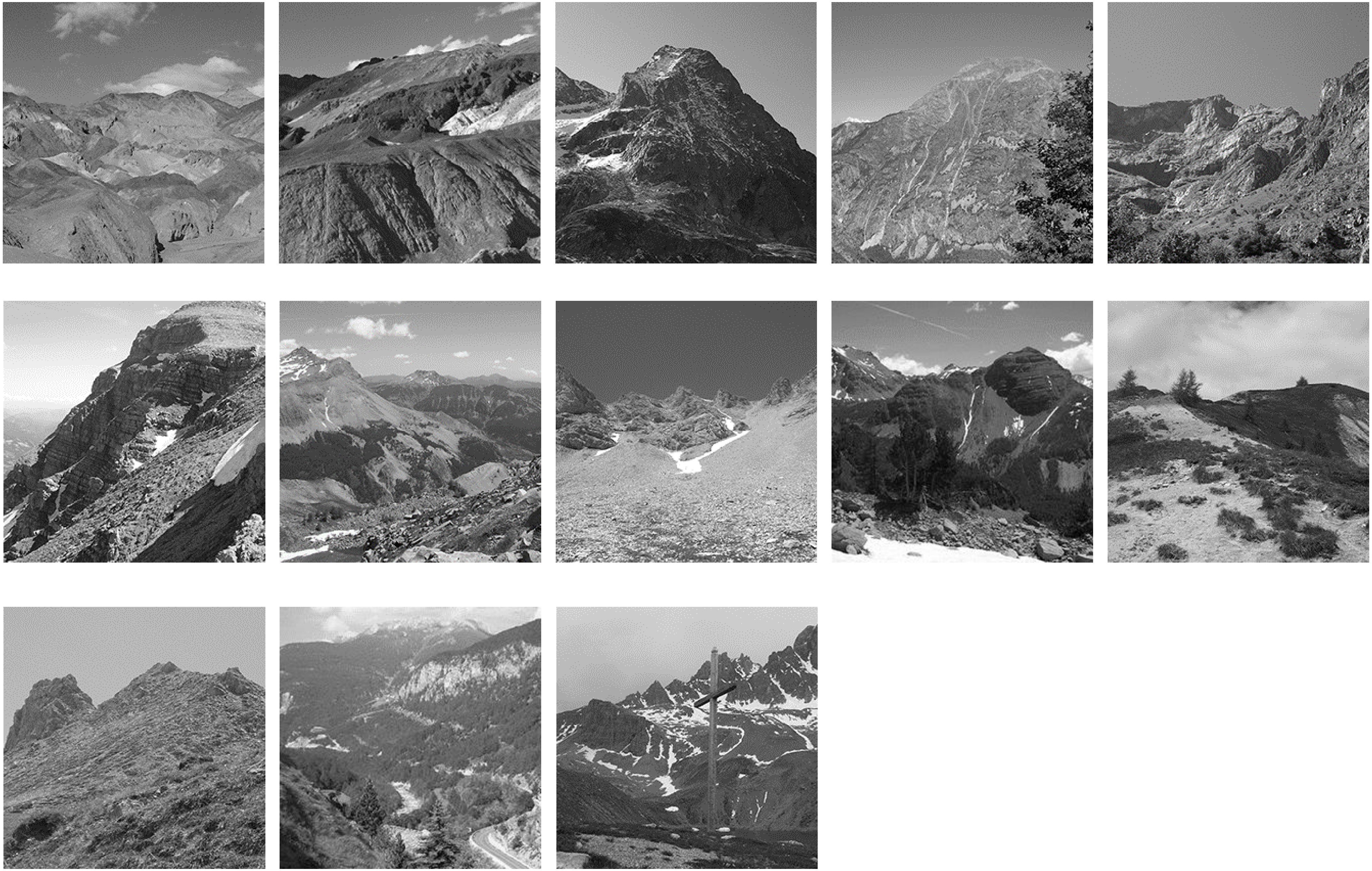

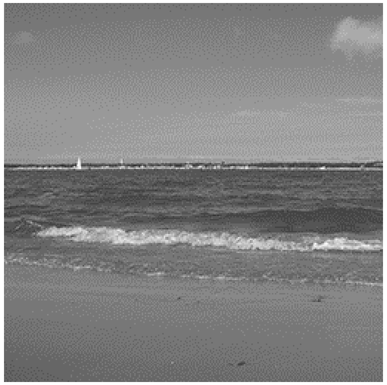
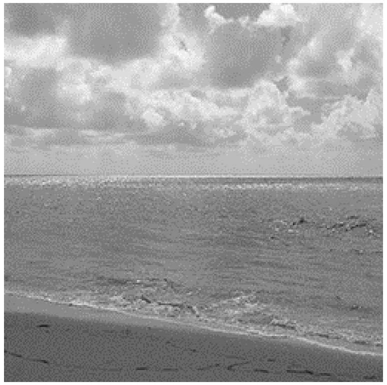
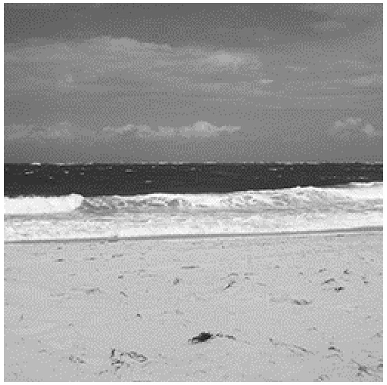
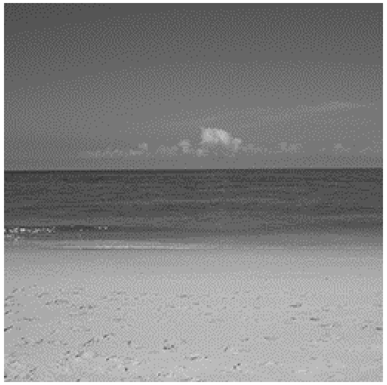
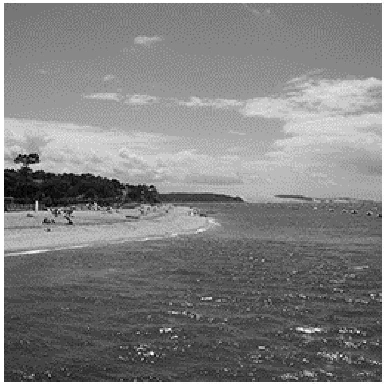
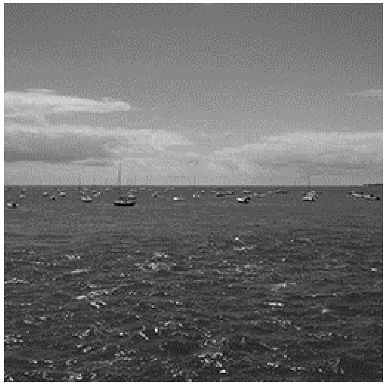
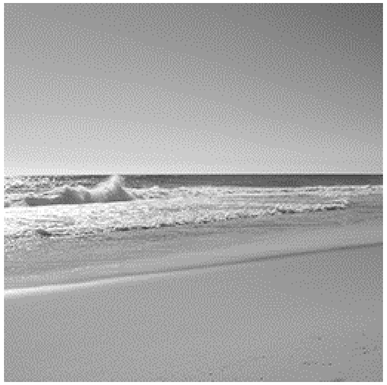
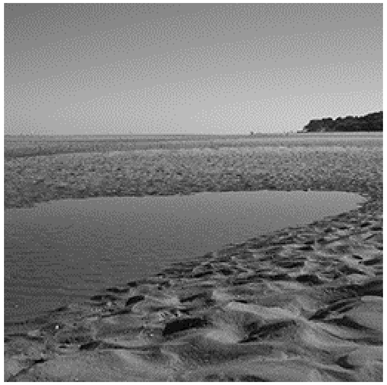
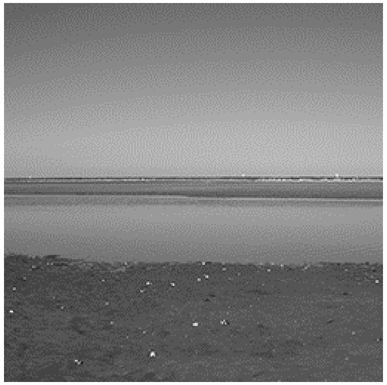
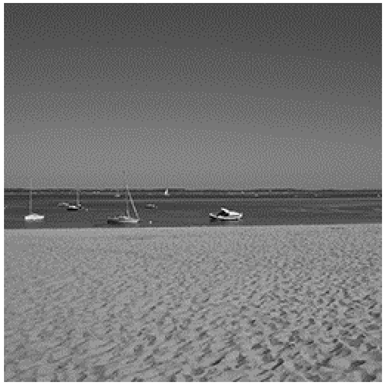
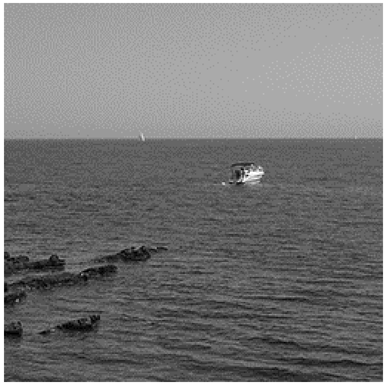
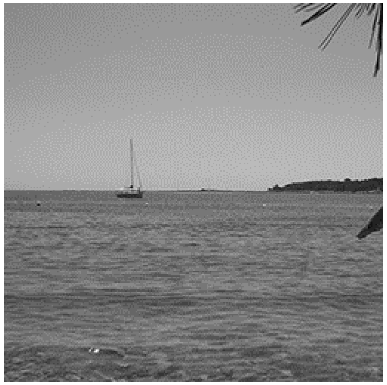
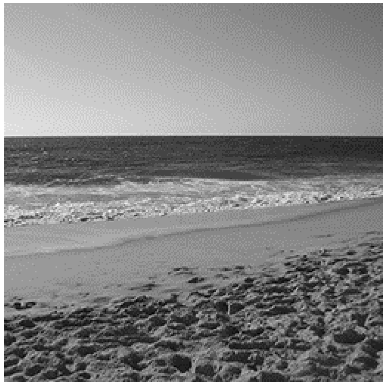

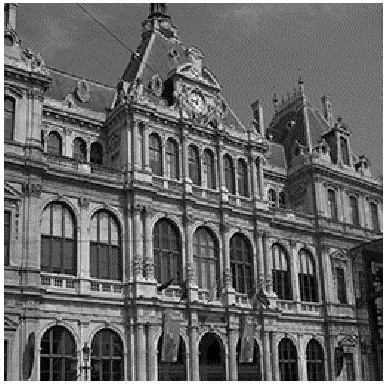
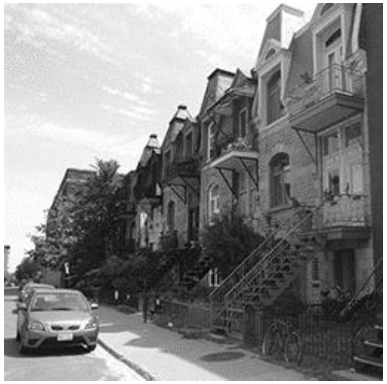
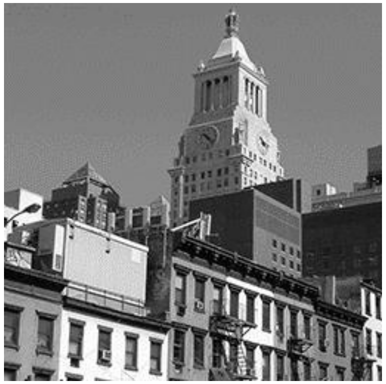
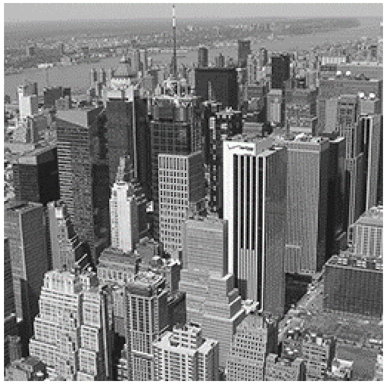
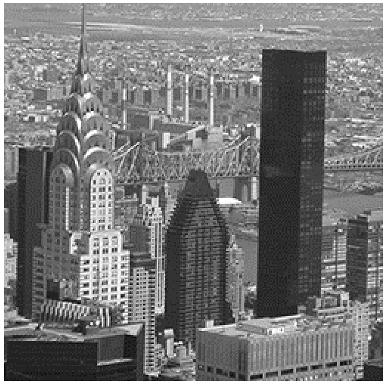
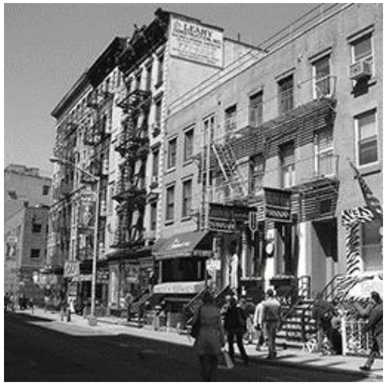
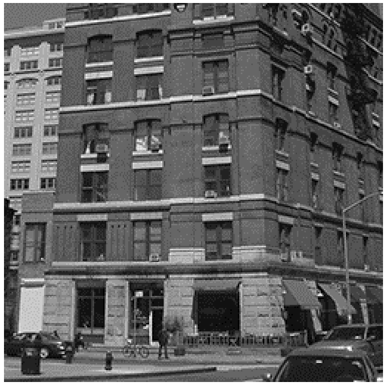
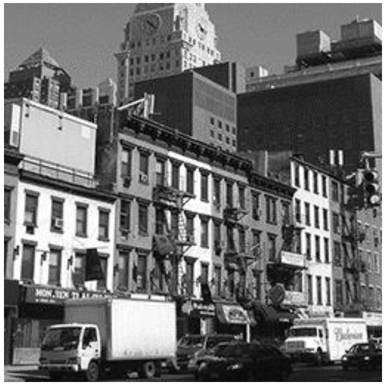
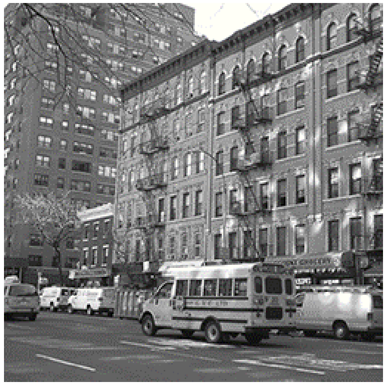
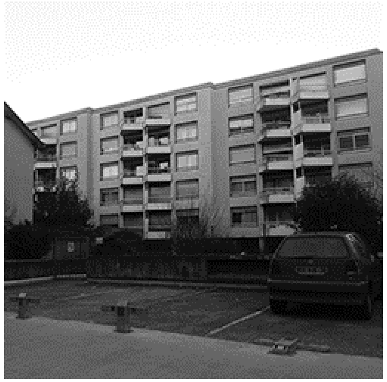
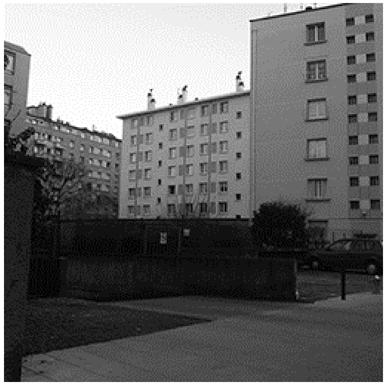
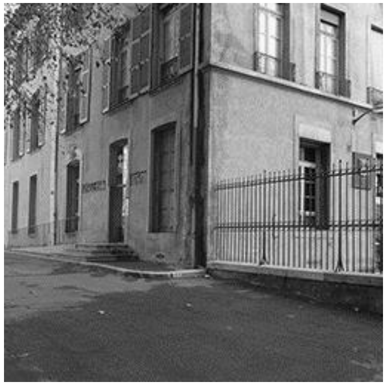
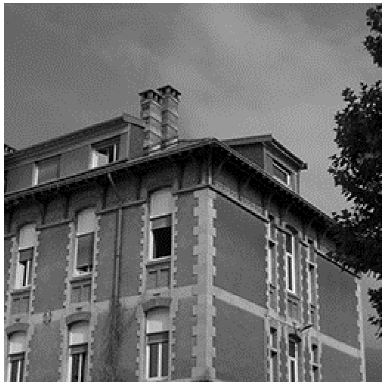

Linear B

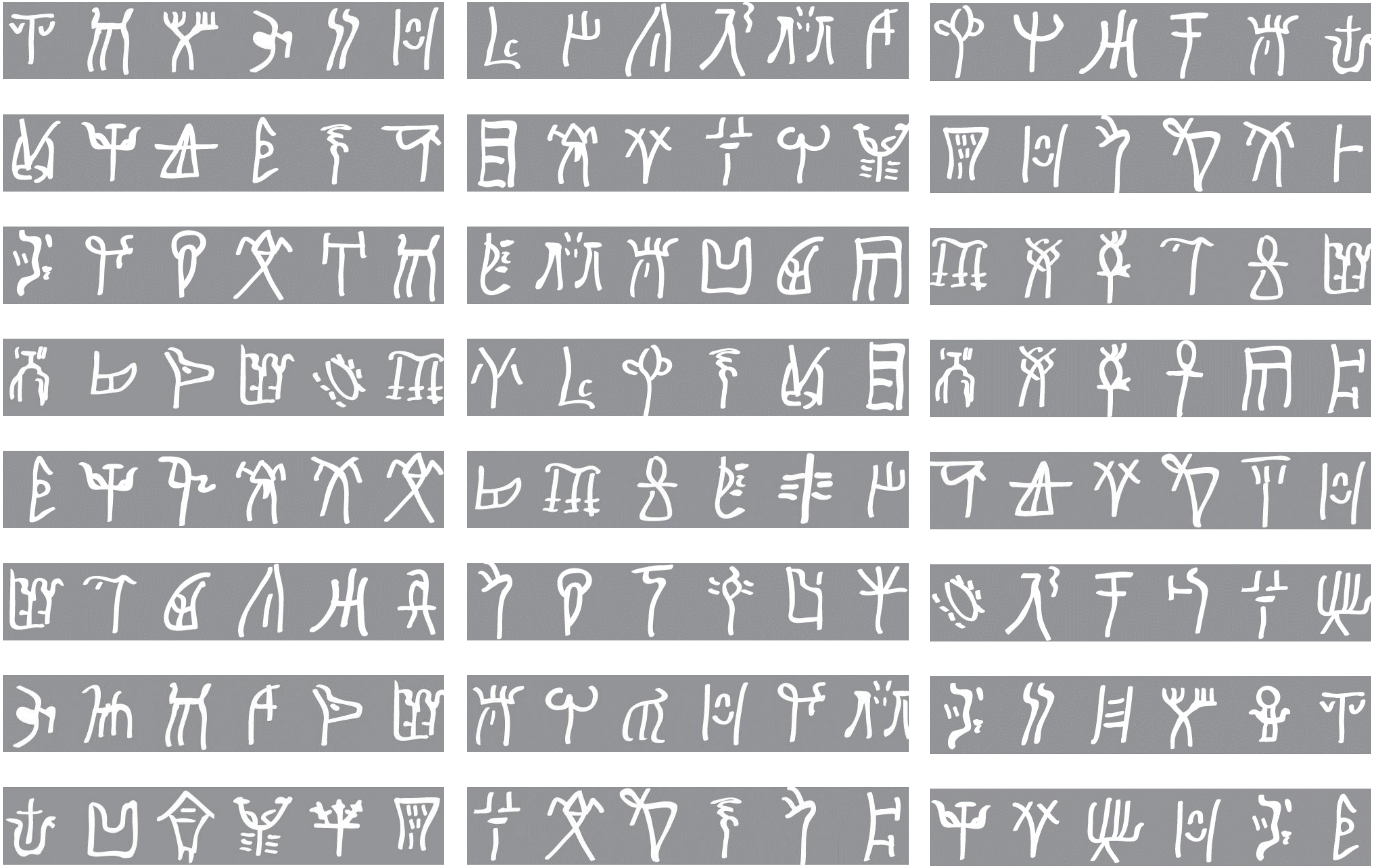

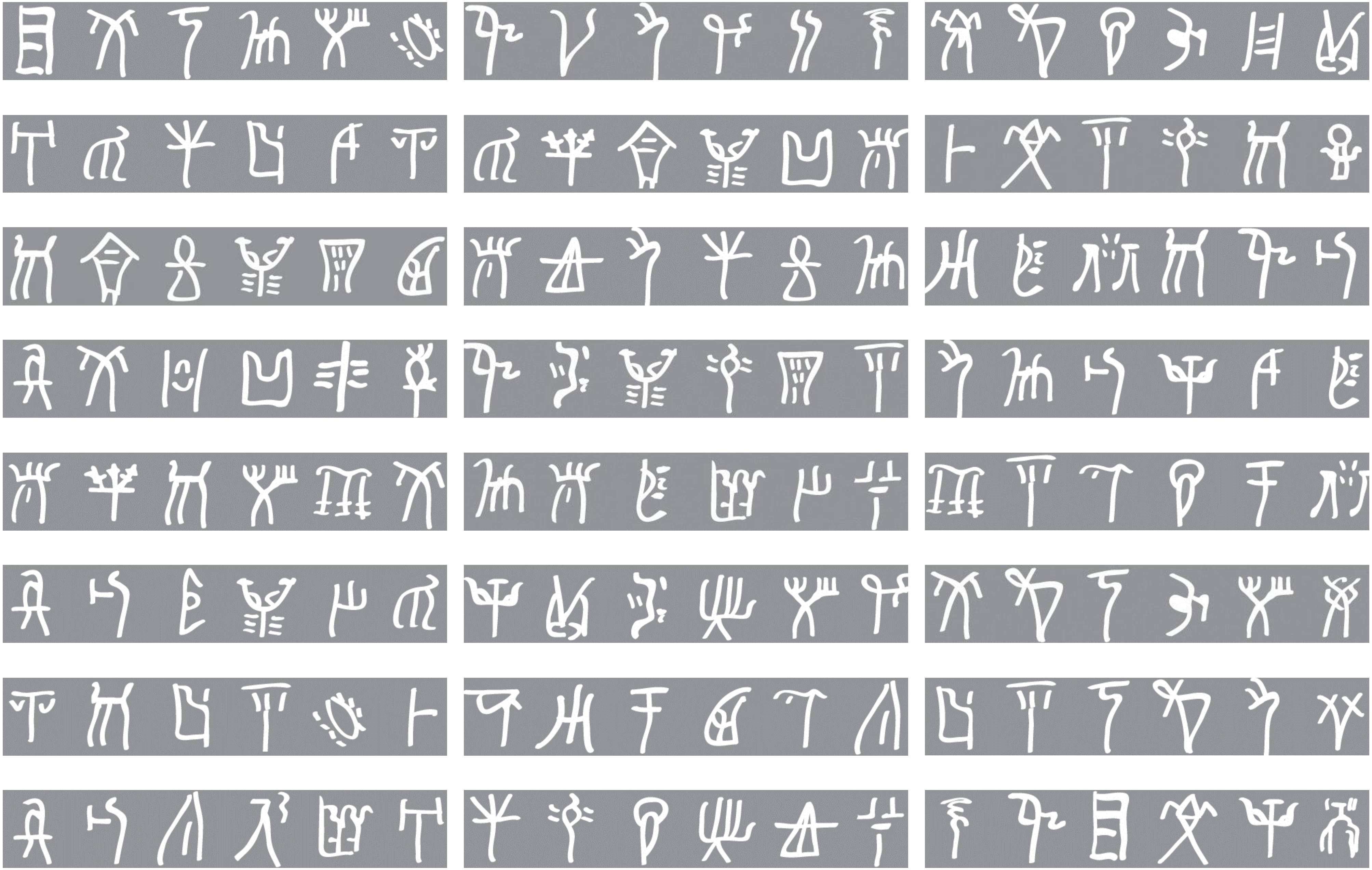

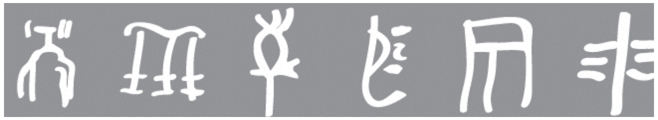
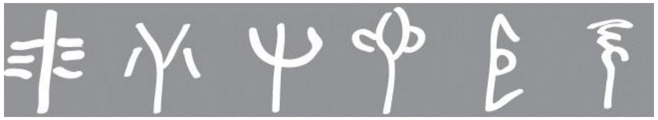
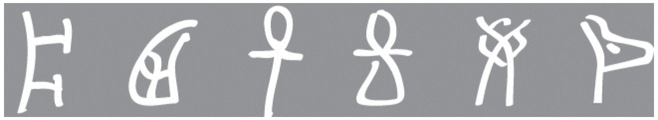
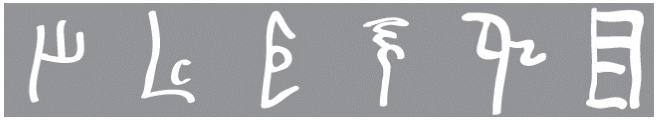

Words

anneau	arcade	audace	aurore	averse
ring	arcade	boldness	dawn	shower
bonnet	calcul	chevet	ciment	ciseau
hat	calculation	bedside	cement	scissors
convoi	cordon	délice	dragon	énigme
convoy	cord	delight	dragon	riddle
espion	farine	flacon	fourmi	hangar
spy	flour	flask	ant	shed
humour	injure	jambon	jument	lézard
humor	insult	ham	mare	lizard
mairie	maquis	moulin	museau	notion
city hall	scrub	mill	muzzle	concept

organe	patate	pigeon	poteau	rafale
organ	potato	pigeon	post	gust
rebord	refuge	relief	remède	renard
edge	refuge	relief	cure	fox
réseau	rivage	roseau	saveur	sirène
network	shore	reed	flavor	siren
tennis	tomate	trajet	tunnel	violon
tennis	tomato	route	tunnel	violin
virage				
bend				
